# Source, Extraction, Properties, and Multifunctional Applications of Pectin: A Short Review

**DOI:** 10.3390/polym16202883

**Published:** 2024-10-12

**Authors:** Le Yi, Lifeng Cheng, Qi Yang, Ke Shi, Fengbo Han, Wei Luo, Shengwen Duan

**Affiliations:** 1 Institute of Bast Fiber Crops, Chinese Academy of Agricultural Science, No. 348 Xianjia Road, Changsha 410205, China; 2 Key Laboratory of Carbohyrate Chemistry and Biotechnology, Jiangnan University, Ministry of Education, No. 1800 Lihu Road, Wuxi 214122, China; 3 Luntai County Star Bo Clothing Co., Ltd., Bazhou 841600, China

**Keywords:** pectin, extraction method, structural characteristics, physical and chemical properties, physiological function

## Abstract

Pectin, a heteropolysaccharide derived from plant cell walls, is essential in the food, pharmaceutical, and environmental industries. Currently, citrus and apple peels are the primary sources for commercial pectin production. The yield and quality of pectin extracted from various plant sources significantly differ based on the extraction methods employed, which include physical, chemical, and biological processes. The complex structures of pectin, composed of polygalacturonic acid and rhamnogalacturonan, influence its physicochemical properties and, consequently, its functionality. As a common polysaccharide, pectin finds applications across multiple sectors. In the food industry, it acts as a gelling agent and a packaging material; in pharmaceuticals, it is utilized for drug delivery and wound healing. Environmentally, pectin contributes to wastewater treatment by adsorbing pollutants. Current research focuses on alternative sources, sustainable extraction methods, and multifunctional applications of pectin. Ongoing studies aim to enhance extraction technologies and broaden the applications of pectin, thereby supporting sustainable development goals.

## 1. Introduction

Pectin is a heterogeneous polysaccharide found between adjacent cell walls and in the middle lamella of plants, comprising carbon (C), hydrogen (H), and oxygen (O) atoms. It was first extracted by the French chemist Braconnot in 1825, who named this gelling substance “pectin” [1]. Since then, numerous researchers have directed their attention to pectin [2]. Currently, global demand for pectin has risen significantly, but its production remains limited. Although most plants contain pectin and materials are abundant, the utilization rate of pectin resources is low, and the pectin extraction industry is inadequate [3]. As a result, a substantial amount of pectin-rich raw materials is discarded, leading to resource waste and environmental pollution. Therefore, comprehensive utilization of pectin raw materials aligns with the principles of “green, ecological, and environmentally friendly” practices. Promoting the extraction and utilization of pectin from unconventional raw materials, as well as exploring new technologies and methods to enhance pectin yield and quality, is crucial for advancing the pectin industry.

## 2. Sources of Pectin

**Figure 1:**
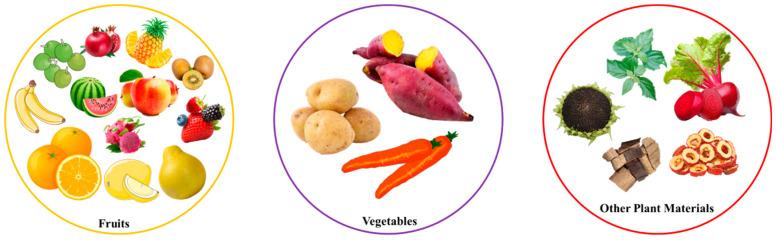
Typical plant materials deriving pectin.

Natural sources of pectin are not only abundant but also diverse, showcasing a wide range of concentrations across various plant-based materials (Figure 1), exhibiting varying concentrations in plant-based materials such as citrus peels, apple peels, and potato residues (Table 1). Currently, commercial pectin is predominantly extracted from citrus fruits and apples [4,5]. Pectin derived from alternative sources remains largely confined to laboratory-scale research [6,7].

In recent years, the potential for using agricultural by-products and food waste, such as pomace from fruit juices or peels from root vegetables, highlights the environmental benefits of pectin extraction, promoting sustainability and reducing overall waste. Overall, the exploration of diverse natural sources for pectin not only broadens the spectrum of applications but also supports innovative approaches to sustainable resource utilization.

## 3. Extraction Methods for Pectin

Pectin exists in three forms: protopectin, pectin, and pectic acid [29]. Pectin predominantly manifests as water-insoluble protopectin and pectic salts within plants. While water-insoluble pectin can dissolve in solutions of organic or inorganic acids, the insoluble pectin found in raw materials can be transformed into soluble pectin. This transformation enables dispersion into the extraction medium through various physical, chemical, and biological methods. The subsequent separation and purification yield high-quality pectin [30]. Generally, pectin extraction methods can be categorized into physical, chemical, and biological approaches, each exhibiting distinct characteristics (Table 2).

### 3.1. Physical Methods

#### 3.1.1. Microwave Method

Microwave radiation is a form of electromagnetic wave with frequencies ranging from 300 MHz to 300 GHz. Microwaves can penetrate extraction media and interact with the polar components of materials, converting them into thermal energy through ionic conduction and dipole rotation mechanisms. The generated heat accelerates mass transfer, facilitating the breakdown of complex cross-linked structures among pectin, cellulose, and hemicellulose, which enhances the dissolution and release of pectin [10]. Spinei et al. utilized microwave technology to extract pectin from grape pomace, identifying the optimal extraction conditions of 560 W microwave power, a pH of 1.8, and an extraction duration of 120 s, resulting in a pectin yield of 11.23% [27]. Prakash Maran et al. employed microwave methods to extract pectin from zucchini peels, achieving the highest yield (25.79%) under the conditions of 477 W microwave power, 128 s of irradiation, a pH of 1.52, and a solid-to-liquid ratio of 1:20.3 g/mL [39]. Therefore, optimizing the power and duration of microwave extraction for different types of plant materials is crucial to ensure the yield and quality of pectin.

Microwave extraction methods not only enhance the yield of pectin but also offer a more economical alternative to traditional extraction techniques. The rapid heating associated with microwave radiation significantly reduces processing time, leading to lower energy consumption and decreased labor costs. Moreover, the stability of pectin structures during microwave extraction is heavily influenced by the extraction conditions. When conditions are optimized, high temperatures can improve the solubilization of pectin, while preserving its functional properties, thereby preventing degradation that may occur with prolonged exposure to conventional heating methods.

#### 3.1.2. Ultrasonic Method

The cavitation effect, shear force, and their combined effects produced by ultrasonic waves collectively exert powerful energy in the solution, disrupting the structure of plant cell walls and enhancing solvent permeation, thereby facilitating the complete dissolution and release of pectin. Lasunon et al. employed ultrasonic-assisted methods to extract pectin from industrial tomato waste, determining that the optimal process parameters were an extraction temperature of 180 °C, an extraction time of 20 min, a pH of 1.5, and a solid-to-liquid ratio of 1:30, resulting in a maximum pectin yield of 34.06% [40]. Dranca et al. extracted pectin from apple pomace using ultrasonic extraction methods, and their optimization results indicated that under an ultrasonic frequency of 100%, a pH of 1.8, a solid-to-liquid ratio of 1:10 (g/mL), and a duration of 30 min, the highest pectin yield achieved was 9.18%, along with a galacturonic acid content of 98.13 g/100 g and an esterification degree of 83.2% [11]. Ultrasonic frequency is among the most significant factors affecting extraction efficiency, and the optimal ultrasonic conditions can be selected in conjunction with ultrasonic time.

Ultrasonic extraction methods offer significant economic advantages by substantially reducing both processing time and energy consumption compared to traditional extraction techniques. The efficiency of ultrasonic waves facilitates the rapid disruption of plant cell structures, leading to more effective solvent permeation and higher pectin yields in shorter durations. Optimized ultrasonic parameters can enhance the solubilization of pectin, while minimizing degradation, thereby ensuring the retention of its functional properties.

#### 3.1.3. Aqueous Two-Phase Extraction Method

Aqueous two-phase extraction is a method for separating mixtures by exploiting variations in component solubility within a solvent. The primary extraction techniques employed for pectin extraction include supercritical fluid extraction, continuous counter-current extraction, and aqueous two-phase extraction. Among these methods, supercritical CO_2_ extraction is the most widely utilized due to its low cost, non-toxic nature, and lack of environmental pollution. Zou et al. investigated the extraction of pectin from hawthorn fruits using supercritical CO_2_ at a temperature of 35 °C, a pressure of 15.0 MPa, and a CO_2_ flow rate of 40 L/h, achieving a maximum pectin extraction rate of 3.1% [41]. Xie et al. applied both continuous counter-current extraction and batch extraction methods to extract pectin from citrus peels, obtaining extraction rates of 8.72% and 6.57%, respectively [42]. The advantages of supercritical CO_2_ extraction stem from its utilization of carbon dioxide as a solvent, which is both cost-effective and recyclable. Furthermore, the ability to precisely control temperature and pressure during the supercritical CO_2_ extraction process allows for the extraction to be conducted without compromising the molecular structure of pectin, thus preserving its bioactivity and functionality.

Commonly used aqueous two-phase systems comprise polymer-based systems, polymer/inorganic salt systems, low molecular weight organic/inorganic salt systems, and surfactant-based systems. Hu et al. utilized a two-phase system to isolate pectin from navel orange peels, achieving the optimal conditions of 35% ethanol volume fraction, 18% dipotassium phosphate, and an extraction temperature of 78 °C, resulting in a pectin yield of 8.35% [43]. The operational conditions of aqueous two-phase systems are mild and exhibit good biocompatibility, reducing the likelihood of denaturing active ingredients, thereby making them suitable for the extraction of bioactive substances. Aqueous two-phase extraction not only benefits from its ability to selectively isolate desired compounds, but also proves economically advantageous due to lower operational costs and reduced solvent waste compared to traditional methods. This economic efficiency is particularly relevant in large-scale industrial applications.

#### 3.1.4. High-Pressure Pulsed Electric Field Method

High-pressure pulsed electric field (HPEF) technology utilizes high-voltage pulses to induce significant damage to plant cell walls and membranes, thereby facilitating the release of target substances from the system [44]. Additionally, the controlled electric field application helps maintain the integrity of pectin’s molecular structure, thereby reducing the likelihood of degradation during extraction. Wang et al. extracted pectin from okra using HPEF, identifying the optimal conditions as a solid-to-liquid ratio of 1:28 (g/mL), an enzyme amount of 600 U/g, a pH of 5.5, an extraction temperature of 53 °C, an electric field strength of 10 kV/cm, nine pulses, and an enzymatic hydrolysis time of 20 min, achieving a pectin yield of up to 17.62% [33]. Gao et al. employed a combination of pulsed electric field and cellulase hydrolysis to extract pectin from grapefruit peels, achieving a maximum pectin yield of 12.08% [45].

#### 3.1.5. Steam Explosion Method

Steam explosion is a method that subjects extraction materials to a high-temperature and high-pressure environment, allowing the pores of the materials to fill with steam. Upon instantaneous release of the high pressure, the liquid within these pores rapidly vaporizes due to the pressure drop, resulting in dramatic volume expansion that ruptures the cells, thereby promoting the release of intracellular pectin. Steam explosion offers an efficient extraction approach that can reduce the processing time and costs associated with traditional extraction methods. The high yields obtained from a relatively short extraction time contribute to enhanced productivity. Liang et al. applied steam explosion to extract pectin from passion fruit peels, achieving a pectin yield of 10.7% [34]. The moisture content of the materials and the grinding fineness significantly influence the effectiveness of the steam explosion; thus, the pretreatment of raw materials is a critical step.

#### 3.1.6. Subcritical Water Extraction Method

Subcritical water is defined as water at temperatures ranging from 100 °C to 374 °C and pressures between 0.1 MPa and 22.1 MPa. By controlling the temperature and pressure of subcritical water, its polarity can be adjusted across a wide range, facilitating the selective extraction and separation of substances continuously. Cui et al. utilized subcritical water to extract pectin from persimmon peels, achieving the optimal extraction conditions of 138 °C, 2.84 min, and a solid-to-liquid ratio of 1:10.02, resulting in a yield of 7.62% [35]. Since it utilizes water, an abundant and inexpensive resource, this technology minimizes the need for costly organic solvents and reduces overall operational expenses, thereby being recognized as a green and environmentally friendly method with significant potential.

### 3.2. Chemical Methods

#### 3.2.1. Acid Extraction Method

Acid extraction methods utilize acids that can be classified as either inorganic acids (e.g., sulfuric acid, hydrochloric acid, nitric acid) or organic acids (e.g., citric acid, acetic acid). These acids hydrolyze the complex cross-linked network in cell walls, thereby promoting the dissolution and release of pectin [46]. Inorganic acids can cause equipment corrosion and environmental pollution; thus, there is a growing trend in acid extraction to seek suitable organic acids as alternatives to inorganic acids for pectin extraction. Jarrín-Chacón et al. reported that the pectin extraction yields from cocoa pods using citric acid, malic acid, and fumaric acid were 6.17%, 5.11%, and 3.2%, respectively [47]. Tian et al. used acid to extract pectin from hawthorn under the conditions of a solid-to-liquid ratio of 1:3, a citric acid solution concentration of 0.1 mol/L, a pH of 2, and a temperature of 55 °C for 20 min, yielding 33.86% pectin with an esterification degree of 45.58% [48]. Pectin undergoes hydrolysis under acidic conditions. Compared to inorganic acids, organic acids are more accessible, biodegradable, exhibit weaker acidity, and contribute less to environmental pollution, making them more suitable for large-scale pectin production. The mild extraction conditions associated with organic acids help maintain the stability of pectin’s molecular structure, minimizing the degradation that may occur under the harsher conditions typical of inorganic acid extraction. These findings suggest that organic acids may serve as promising green solvents for pectin extraction, as opposed to inorganic acids.

#### 3.2.2. Ammonium Oxalate Method

Ammonium oxalate can convert insoluble calcium pectate into soluble pectic acid, with calcium ions combining with oxalate ions to form calcium oxalate precipitate [49]. Ammonium oxalate exhibits strong metal chelating properties, enabling it to chelate calcium ions in calcium pectate, thereby forming soluble ammonium salts and increasing the solubility of pectin under relatively mild treatment conditions. Li et al. utilized ammonium oxalate to extract pectin from pumpkins, achieving an average pectin yield of 6.09% [50]. Cheng et al. employed the ammonium oxalate extraction method to extract pectin from ramie bast fiber, determining that the optimal process parameters included an extraction temperature of 90 °C, a solid-to-liquid ratio of 1:30, an ammonium oxalate concentration of 0.95%, and an extraction time of 4 h, resulting in a yield of 15.81% [51]. However, the ammonium oxalate method has certain limitations, such as the potential introduction of oxalate ions during the extraction process. This may require additional steps to eliminate these ions to prevent adverse effects on the properties of pectin.

#### 3.2.3. Ion Exchange Method

In the ion exchange resin extraction method, cationic resins containing H^+^ can exchange cations, such as Ca^2^^+^ and Mg^2^^+^, in pectin, thereby releasing the bound cations and accelerating the dissolution of pectin, which increases the overall pectin yield [52]. Liu et al. extracted pectin from pumpkin peels using ion exchange methods, determining that the optimal conditions were an extraction time of 2.5 h, an extraction temperature of 80 °C, a pH of 2.0, and a solid-to-liquid ratio of 30:1 (mL/g), with a resin dosage of 5%, resulting in a pectin yield of 18.5% [53]. The resins interact with calcium and magnesium ions from the plant materials, disrupting their ionic bonds with pectin through exchange reactions. Additionally, the resins can adsorb small molecular substances, eliminating the mechanical entanglements of pectin and reducing the minimum energy required for the extraction of pectin from plant materials, thereby significantly enhancing the pectin yield.

### 3.3. Biological Methods

#### 3.3.1. Microbial Methods

Microbial fermentation produces enzymes that liberate pectin from plant tissues by selectively degrading the complex polysaccharides present in plant materials, thereby effectively extracting pectin from these tissues. Huang utilized microbial fermentation to extract pectin from citrus peels, identifying the optimal conditions as a 10% inoculum, a fermentation time of 40 h, a fermentation temperature of 36 °C, a substrate pH of 7.0, and the addition of 1% urea, achieving a maximum pectin extraction rate of 21.5% [54]. Liu et al. [38] extracted pectin from persimmon peels through fermentation with *Aspergillus terreus*, demonstrating that the optimal fermentation conditions included a fermentation time of 30.09 h, a fermentation temperature of 25 °C, and an initial pH of 6.9 for the fermentation medium, resulting in a maximum pectin yield of 0.449 g/g (weight of the extracted pectin/weight of the raw material). Notably, pectin extracted via microbial fermentation methods has a relatively high molecular weight, low chemical reagent consumption, and minimal environmental pollution, along with high gelling properties and stable quality [55].

From an economic perspective, microbial fermentation presents a cost-effective and sustainable method for pectin extraction, as it requires fewer chemical reagents and depends on natural microbial processes. This approach not only reduces input costs associated with traditional extraction techniques but also enhances overall productivity due to the higher yields achieved. Furthermore, the mild fermentation conditions help maintain the stability of pectin’s molecular structure, minimizing degradation and preserving its functional characteristics. This is particularly advantageous for applications requiring high-quality pectin. Thus, microbial fermentation emerges as a promising and environmentally friendly method for extracting pectin from various plant sources.

#### 3.3.2. Enzymatic Methods

Enzymatic extraction methods commonly employ a combination of proteases, cellulases, hemicellulases, and ligninases to hydrolyze plant cell walls, facilitating the release of pectin. Wikiera et al. extracted pectin from apple pomace using endo-xylanase under the conditions of 40 °C and pH 5.0, with shaking for 10 h. The efficiency of pectin extraction using endo-xylanase reached 19.8%, and the resulting polymer exhibited a high molecular weight. Endo-xylanase degrades xylan by cleaving the β-1,4-glycosidic bonds within the xylan molecules, loosening the cell wall structure and aiding in the release of pectin bound to xylan. The use of this enzyme alone demonstrated the highest pectin extraction efficiency, indicating that xylan degradation is crucial for the release of pectin [55]. Dai et al. employed an ultrasound-assisted composite enzyme method to extract pectin from tangerine peels using a combination of hemicellulase and cellulase. Under the conditions of a solid-to-liquid ratio of 1:20 (g/mL), ultrasound power of 187.5 W, ultrasound duration of 65.3 min, a composite enzyme amount (*m*_hemicellulase_:*m*_cellulase_ = 1:1) of 5.6 mg, pH 4.82, and a temperature of 41.8 °C, the pectin yield was 11.93% [56]. Compared to single enzyme methods, the composite enzyme method reduced processing time by 4 to 5 h and improved extraction efficiency. Ezzati used ultrasound-assisted extraction technology to extract pectin from sunflower by-products, achieving optimal extraction conditions at an ultrasound duration of 30 min, a temperature of 33 °C, and power of 400 W, resulting in an extraction rate of 11.15% [17]. Megías-Pérez et al. utilized endo-xylanase and endo-cellulase to extract pectin from apple pomace, finding that endo-xylanase yielded the highest extraction rate of 19.8%; while, pectin extracted using endo-cellulase had a lower molecular weight [26]. Generally, biological enzymes are prone to denaturation and deactivation, typically requiring temperatures around 50 °C and a pH of 3.5 to 4.5 for pectin extraction.

Enzymatic extraction methods provide a cost-effective alternative to traditional extraction processes, as they utilize lower energy inputs and fewer harsh chemicals. The combination of enzymes can lead to higher extraction yields and decreasing processing times, thereby optimizing resource use. Moreover, the extraction conditions are relatively mild, so it helps maintain the stability of pectin’s molecular structure, reducing the risk of degradation that can occur with harsher treatments.

In pectin extraction, physical, chemical, and biological methods each have their unique characteristics and wide-ranging applications. Moreover, the efficiency of pectin extraction is influenced by various factors, such as extraction conditions, the properties of plant materials, and the techniques employed. Therefore, future research could focus on the combination of multiple extraction methods to achieve higher yields and better-quality pectin products. Additionally, exploring novel green solvents and optimizing extraction processes will further advance the application of pectin.

## 4. Structure and Physicochemical Properties of Pectin

### 4.1. Structure of Pectin

**Figure 2:**
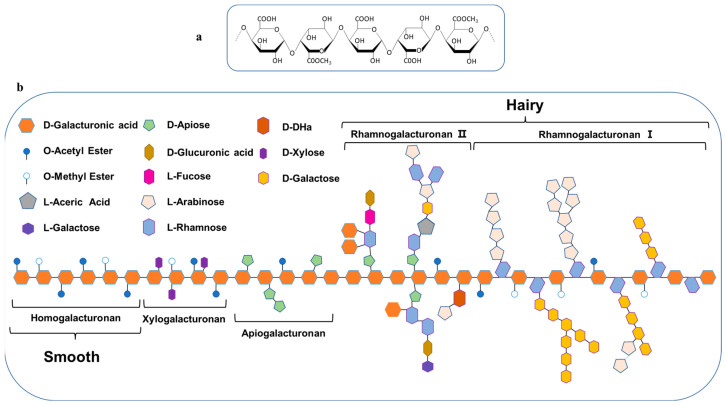
Schematic structure of pectin [59]. (**a**) Linear chain; (**b**) branched chain. D-DHA: 3-deoxy-D-lyxo-heptulosaric acid.

Pectin is a natural polysaccharide located in plant cell walls, primarily extracted from materials such as citrus peels, apples, and sunflowers. This complex polysaccharide comprises long chains of α-(1,4)-D-galacturonic acid (Figure 2a), some of which may be partially methylated, meaning that certain carboxyl groups of galacturonic acid are substituted with methoxy groups. Pectin can be categorized into four components: polygalacturonic acid, rhamnogalacturonan-I, rhamnogalacturonan-II, and xylogalacturonan [57]. These polymers are interconnected by covalent bonds. The high-resolution model of pectin illustrates the widespread presence of these four components (Figure 2b). Although the composition and quantity of each component vary among different sources of pectin, rhamnogalacturonan-I and polygalacturonic acid are the primary components; while, rhamnogalacturonan-II and xylogalacturonan serve as secondary components. The characteristic feature of xylogalacturonan (XG) is that some of the galacturonic acid residues in its main chain have xylose attached at the C-3 position [58]. Variations within pectin are particularly noted in the side chains of rhamnogalacturonan-I. Additionally, two rhamnogalacturonan-II units containing celery sugar residues are linked through a boric acid ester to form a rhamnogalacturonan-II dimer [59]. The main structure of the “smooth” pectin region consists of a linear and partially esterified HG backbone, which accounts for approximately 60% of the total pectin. The “hairy” regions are composed of highly branched RG-I and RG-II domains, along with smaller quantities of XG and AG.

### 4.2. Physicochemical Properties of Pectin

Pure pectin is a light-yellow powder that is odorless. It dissolves in 20 times its weight of water, yielding a viscous liquid, and is insoluble in organic solvents. Pectin extracted from various plant materials and through different extraction processes demonstrates significant variations in physicochemical characteristics, including the degree of esterification, gelling ability, and emulsifying properties (Table 3).

#### 4.2.1. Esterification Degree (DE)

The degree of esterification (DE) of pectin refers to the ratio of methoxylated galacturonic acid units to the total galacturonic acid units present. Based on the DE, pectin can be classified into high-ester pectin (DE ≥ 50%) and low-ester pectin (DE < 50%) [6]. The DE serves as a critical indicator of pectin properties, influencing its structure and conformation [61]. There are notable structural and conformational differences in pectin depending on its DE; as the DE decreases, the flexibility of the pectin molecular chain diminishes; while, its rigidity increases. Furthermore, an increased DE can lead to reduced extensibility and coiling of pectin molecules, along with an increase in the hydrating volume [62]. The degree of esterification (DE) of pectin not only affects its mechanical stability but also influences its functional properties in gelation and emulsification environments.

#### 4.2.2. Gelling Ability

As a colloid, pectin can form a gel in aqueous solutions under certain conditions, demonstrating excellent gelling properties. Pectin is classified into high-methoxyl pectin (HMP) and low-methoxyl pectin (LMP) based on its methoxy content. Pectin with a methoxy mass fraction ranging from 7% to 16.32% is designated as high-methoxyl pectin; whereas, pectin with a methoxy content below 7.0% is classified as low-methoxyl pectin [63]. High-methoxyl pectin can form gels at specific sugar concentrations, pH levels, and elevated temperatures (gelling upon slight cooling); while, low-methoxyl pectin can gel in the presence of Ca^2^^+^ or other cations, regardless of sugar presence. The higher the degree of methoxylation of pectin, the greater its molecular weight and gelling ability [61]. Pectin serves a vital role as a gelling agent in products ranging from jams to yogurts. Its ability to create stable gels enhances texture and mouth feel, making it an indispensable ingredient. In emulsion-based products, the emulsifying properties of pectin can stabilize mixtures and influence shelf life, fatty acid profiles, and textural attributes.

#### 4.2.3. Emulsifying Capacity

Pectin, owing to its hydrophilic carboxyl groups and hydrophobic methyl and acetyl groups, exhibits notable emulsifying properties. Yang et al. demonstrated that potato pectin possesses strong emulsifying ability, with its emulsifying capacity comparable to that of soybean soluble polysaccharide emulsions in terms of particle size. Additionally, the emulsifying ability increases with the incorporation of pectin [13].

The structure of pectin, characterized by its complex polysaccharide composition, is crucial for various applications ranging from food to pharmaceuticals. Understanding the relationship between its structural components and physicochemical properties is essential for optimizing its utility. Furthermore, future research should investigate modifications in pectin extraction processes to enhance specific properties for targeted applications.

## 5. Purification Methods for Pectin

Following extraction, pectin dissolves in the liquid phase, yielding an extract that necessitates further separation to isolate the target pectin substance, utilizing its physical and chemical properties. The purification techniques employed can significantly influence pectin’s chemical composition, molecular weight distribution, average molecular weight, and gelling capability. The most prevalent separation and purification methods, both domestically and internationally, include alcohol precipitation, salting-out, and membrane separation. Huang utilized alcohol precipitation and salting-out to isolate pectin from citrus peel waste, determining that the optimal conditions for alcohol precipitation involved an ethanol solution concentration of 75%, a temperature below 45 °C, and a precipitation duration of 60 min. For salting-out, the optimal parameters consisted of 5 mL of saturated ferric chloride solution per 50 mL of pectin extract, at a temperature of 60 °C and a precipitation period of 60 min, resulting in derived pectin that met national standards [54]. Yapo et al. found that ultrafiltration purification followed by alcohol precipitation resulted in a reduction in ash and protein content by factors of 1.9 and 4.6, respectively, compared to pectin precipitated alone [64]. Utilizing a membrane with a sufficiently large molecular weight cut-off for ultrafiltration prior to alcohol precipitation effectively removes contaminants, such as ash and proteins, thereby enhancing the quality of the final pectin product and its potential gelling properties.

The purification of pectin is critical, as its purity and characteristics directly impact its application efficacy. Current research employs methods, such as alcohol precipitation, salting-out, and membrane separation, which not only effectively separate and purify pectin but also preserve its biological activity and physical properties during optimization. By precisely controlling the precipitation conditions and parameters, such as alcohol concentration, temperature, and duration, researchers can obtain pectin products that meet quality standards. In the future, with continuous technological advancements and innovations, pectin purification methods are expected to be further optimized, promoting their widespread application in food, pharmaceuticals, and other fields.

## 6. Functions and Applications of Pectin

**Figure 3:**
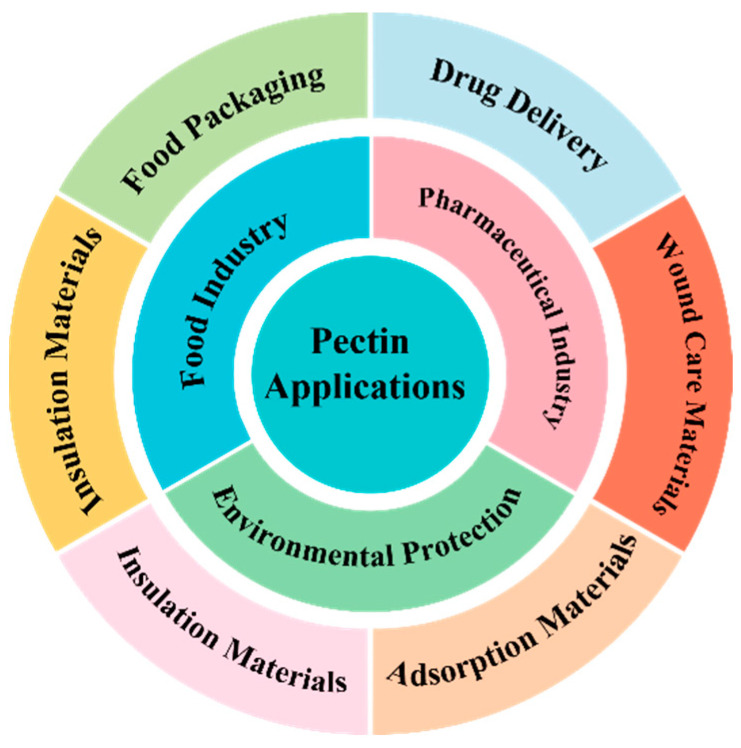
Schematic representation of the pectin application’s classification.

Pectin is non-toxic, harmless, and safe, serving as a primary component of dietary fiber among the seven essential nutrients for the human body. It is also widely recognized as a safe and non-toxic natural food additive [2]. Pectin exhibits several beneficial properties, including gelling, thickening, and stabilizing, which contribute to its extensive applications in the food, pharmaceutical, personal care, and textile industries (Figure 3).

### 6.1. Pectin Applications in Food

#### 6.1.1. Food Additives

Pectin is a green, environmentally friendly natural plant extract recognized as one of the healthiest and safest food additives in nature. It is recommended by the Food and Agriculture Organization of the United Nations and the World Health Organization with no restrictions on addition levels [62]. Its unique gelling properties make pectin widely utilized in jellies, candies, jams, and ice creams to enhance taste and preserve flavor. In dairy products such as yogurt, the superior emulsifying stability of pectin prevents separation and layering. Furthermore, due to its low sugar and low calorie characteristics, pectin can function as a fat substitute in health foods [65], also improving gas retention in dough for baked goods [19].

#### 6.1.2. Food Packaging

Traditional plastic food packaging poses significant environmental and health concerns; thus, researchers are exploring eco-friendly biodegradable alternatives. Pectin has emerged as an ideal choice for food packaging materials due to its biodegradability, biocompatibility, gelling properties, and non-toxicity, making it a promising biobased packaging option [66]. Pectin-based materials can effectively slow respiration and oxidation processes in food, thereby extending its shelf life. Additionally, pectin can function as an edible film or coating, safeguarding the nutritional components of food and preventing undesirable changes, such as loss of color, odor, and flavor, as well as microbial contamination during storage [67].

In the field of food packaging, polysaccharide-derived aerogels are gaining attention for their applications in preservation, maintaining the original flavor of food, and exhibiting antibacterial properties. Kumar et al. [66] developed an enhanced pectin-based nanocomposite film that significantly improves its mechanical properties and antibacterial efficacy by incorporating crystalline nanocellulose and zinc oxide nanoparticles, while ensuring safety for food contact. This film not only demonstrates excellent mechanical strength and barrier properties but also exhibits antibacterial effects against common foodborne pathogens, such as *Staphylococcus aureus* and *Salmonella* spp., thereby meeting established standards for food packaging materials. Researchers have also created a multifunctional active packaging material for strawberry preservation, using pectin as the matrix and incorporating chitosan containing natamycin. This film effectively blocks external UV light, protects natamycin from light degradation, and achieves long-term antibacterial effects within the packaging. Experiments indicate that this active packaging significantly extends the shelf life of strawberries at room temperature [68]. Mao et al. developed a novel pectin-based intelligent active film that integrates Schiff base compounds (SPSs) for monitoring and preserving fruits. The study revealed that SPSs are compatible with the pectin matrix, enhancing the film’s thermal stability, water resistance, and light-blocking capability [69]. The film demonstrates robust antioxidant and antibacterial properties, effectively prolonging the shelf life of cherry tomatoes and fresh-cut mangoes. Additionally, the intelligent film can indicate color changes based on pH fluctuations in the environment, assisting in the monitoring of fruit freshness. Thus, it provides valuable insights for the development of novel intelligent food packaging materials.

#### 6.1.3. Functional Food Ingredients

As a dietary fiber, pectin can lower blood sugar and cholesterol levels, promote gastrointestinal motility, and facilitate bowel movements, thereby contributing to improved gut health. It is also utilized in the development of functional foods and nutritional supplements. Furthermore, pectin possesses significant water absorption and retention capabilities, inducing a sense of fullness after consumption, which reduces food intake and supports weight loss.

In the food industry, its multifaceted roles from additives to packaging illustrate an importance in safety, preservation, and nutrition. The ongoing innovation in pectin-based materials, particularly in active packaging and smart technologies, suggests significant potential for future advancements in food preservation and quality monitoring.

### 6.2. Pectin Applications in Medicine

Pectin demonstrates notable regenerability, gelation, biodegradability, and compatibility, along with various pharmacological and physiological functions, including antibacterial, anticancer, lipid-lowering, and anti-diarrheal properties [70]. It can lower blood sugar and cholesterol levels, inhibit cancer cell aggregation, and prevent metastasis, while also providing cardioprotective effects [71,72].

#### 6.2.1. Drug Delivery

Pectin-based hydrogels are employed as drug carriers due to their biocompatibility and adjustable release profiles. Additionally, pectin can interact with drug molecules to form covalent bonds, ionic bonds, and hydrogen bonds, aiding in the regulation of drug release rates and enhancing bioavailability. Pectin is considered one of the most promising components in colonic-targeted drug formulations due to its specific degradation capability in the colon. It remains stable in the fluctuating gastrointestinal environment and is degraded by pectinase produced by microorganisms in the colon, making it suitable as an oral drug carrier for colonic-targeted delivery [73,74].

Abbasi et al. investigated a novel biodegradable pH-sensitive hydrogel for targeted drug delivery in ulcerative colitis. By copolymerizing pectin with polyethylene glycol (PEG) and methacrylic acid (MAA) through radical polymerization, they successfully prepared an interpenetrating polymer network (IPN). This hydrogel demonstrated targeted drug release in both in vitro and in vivo assessments, significantly improving drug accumulation at the target site, while minimizing the non-target effects of chemotherapy. The study confirmed its biodegradability by the natural microbiota present in the colon, and toxicological experiments indicated its safety in mouse models. This research provides an effective controlled release system for the treatment of ulcerative colitis [75].

**Figure 4:**
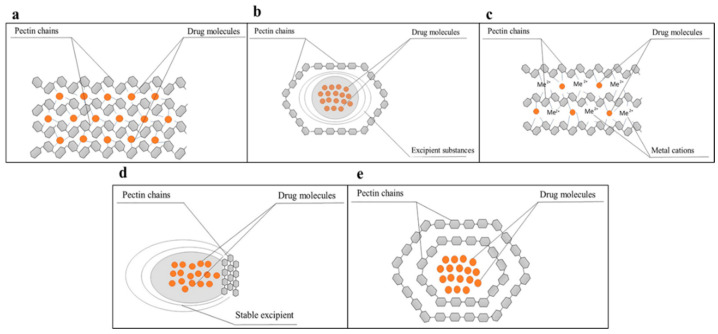
Schematic cross-linking type and mechanism between pectin hydrogels and drug molecules [74]. (**a**) Drugs encapsulated in pure pectin hydrogels; (**b**) drugs combined with microcrystalline fibers and encapsulated within a pectin membrane; (**c**) the “egg box” model of interaction between pectin and divalent cations forms a stable specific structure; (**d**) enzyme-sensitive drug release “bag”; (**e**) pectin nanoparticles containing drugs.

The types and mechanisms of cross-linking between pectin hydrogels and drug molecules are illustrated (Figure 4). Pectin chains can undergo cross-linking through various mechanisms, including interactions with divalent or multivalent cations, aggregation with oppositely charged polyelectrolytes, mixing with viscous polymers and/or calcium salts, or coating with viscous, pH-resistant, or poorly water-soluble polymers. Pectin combines with these therapeutically effective compounds in different ways to form stable structures, ensuring they are protected from degradation in the stomach. These properties can be effectively utilized to develop targeted drug delivery systems [74].

#### 6.2.2. Wound Dressings

Pectin-based hydrogels and films are effective wound dressings, promoting wound healing, while reducing the risk of infection. Hydrogel dressings create a moist wound environment, prevent secondary infections, facilitate the self-debridement of necrotic and granulation tissues, and enhance wound healing efficiency, making them an ideal choice for such applications. Hydrogels composed of pectin and chitosan promote cell proliferation and are safe and non-toxic, making them suitable for use as wound dressings [76].

Researchers have developed a novel pectin/cellulose composite hydrogel prepared through chemical crosslinking for hemostatic applications. The results indicate that the hydrogel possesses a dense network structure, retains the chemical bonds and crystalline structure of both pectin and cellulose, and demonstrates good thermal stability as well as excellent biocompatibility. This hydrogel exhibits outstanding hemostatic effects in both in vitro and in vivo experiments, significantly reducing bleeding in a short period. Consequently, this composite hydrogel holds promising clinical applications as a potential rapid hemostatic wound dressing [77].

Alsakhawy et al. developed an Arabic gum/pectin hydrogel loaded with hesperidin for wound healing [78]. The hydrogel showed high encapsulation efficiency of hesperidin and good physicochemical stability. In animal models, this hydrogel accelerated the wound healing process by promoting angiogenesis, re-epithelialization, and collagen deposition. Additionally, it significantly reduced inflammatory responses and apoptosis, while enhancing antioxidant capacity. Therefore, this hydrogel is recognized as a promising material for wound healing.

Pectin has attracted significant attention within the medical field due to its unique properties, including ease of gelation, biocompatibility, biodegradability, and low cost. It is readily available in various forms, such as hydrogels, films, scaffolds, and nanoparticles. Notably, pectin-based hydrogels can be utilized not only for wound dressings and drug delivery systems, but also in tissue engineering, biosensors, and various other fields of research and application.

### 6.3. Pectin Applications in Wastewater Treatment

#### 6.3.1. Heavy Metal Ion Adsorption

The issue of water source pollution caused by heavy metal ions has garnered significant attention, as these pollutants are challenging to degrade in the environment and pose threats to ecosystems and human health. Singha et al. studied the synthesis of a novel interpenetrating polymer network (IPN) hydrogel for the efficient adsorption and removal of toxic divalent heavy metal ions (such as Pb(II), Cu(II), Co(II), and Zn(II)) from water through solution polymerization [79]. Using a solution polymerization method, monomers (such as sodium acrylate (SA) and N-isopropylacrylamide (NIPA)) are combined with natural polymers (such as pectin (PC)), employing crosslinking agents (like N, N’-methylenebisacrylamide (MBA)) and initiators (such as potassium persulfate (PPS) and sodium bisulfite (SBS)) to synthesize the interpenetrating polymer network (IPN) hydrogel. Under optimized synthesis conditions, this hydrogel exhibited excellent physicochemical properties and recyclability. Adsorption experiments indicated that the maximum adsorption capacities (ACs) of the hydrogel for Pb(II), Cu(II), Co(II), and Zn(II) were 54.86, 53.86, 51.72, and 50.01 mg/g, respectively. These results demonstrate excellent adsorption performance under optimal conditions, with the adsorption process conforming to thermodynamic and kinetic requirements, highlighting its potential application value in water treatment and environmental protection.

#### 6.3.2. Dye Adsorption

Untreated discharge of industrial dyes can disrupt the ecological balance and pose health risks. Singha et al. successfully synthesized a novel interpenetrating polymer network (IPN) hydrogel for the efficient adsorption and removal of harmful dyes from water, including methyl violet, methylene blue, and methyl orange. Adsorption experiments indicated that the hydrogel had maximum adsorption capacities (ACs) of 265.49 mg/g for methyl violet and 137.43 mg/g for methylene blue [79]. The hydrogel demonstrated a high adsorption capacity for these pollutants, with the adsorption process conforming to thermodynamic and kinetic requirements. The successful preparation of such adsorbents is significant in the field of dye pollution treatment and contributes to the development of effective wastewater adsorption materials.

### 6.4. Other Applications

In addition to its applications in food and medicine, pectin is widely utilized in various industries, including papermaking, textiles, and cosmetics, due to its favorable film-forming properties, water retention, radiation resistance, and specific viscosity [80]. In cosmetics, pectin serves as an emulsifier [81]; in microbiological culture media, it can replace agar as a gelling agent; and in the water treatment industry, pectin-based green scale inhibitors can be developed to prevent the formation of calcium sulfate [82]. Moreover, pectin is being explored in the development of new biodegradable biomaterials, serving as a matrix for printing materials in 3D printing [83,84] and for its potential application in active packaging materials [67,85]. In agriculture, pectin acts as a soil conditioner, improving soil structure and enhancing water retention and fertility. Additionally, pectin-based materials, due to their stability and biocompatibility, are being employed to develop biosensors for detecting chemicals or biomolecules in the environment.

Pectin showcases a remarkable versatility due to its unique physicochemical properties, which facilitate its diverse applications across multiple fields. As consumer demand shifts towards sustainable and safe products, pectin represents a promising candidate for addressing both health and environmental challenges. As research continues to explore and expand pectin’s capabilities, it will likely emerge as a critical component in the development of new products and technologies that align with health, safety, and environmental sustainability objectives.

## 7. Future Perspectives

Pectin, a natural polysaccharide derived from plant sources, provides numerous nutritional and safety benefits alongside a variety of bioactive properties. This diversity facilitates a multitude of potential applications. The widespread availability of pectin from various botanical sources, combined with diverse extraction techniques, presents significant opportunities for innovation. It is essential to investigate the sustainable development and utilization of pectin sourced from agricultural by-products, as this approach could enhance the economic value of these materials, while supporting initiatives for rural revitalization.

Nevertheless, the pectin industry currently faces two major challenges: (1) Environmental pollution and high energy consumption that is linked to traditional extraction methods. The frequent use of strong acids and bases in pectin extraction can result in adverse ecological effects and the potential degradation of pectin’s structural integrity. Transitioning to biological extraction methods utilizing microbial and enzymatic preparations may effectively alleviate these issues and promote environmental sustainability. (2) Limited diversity in raw material sources remains a challenge. Currently, commercial pectin is predominantly extracted from citrus and apple peels, underscoring the urgent need for industrial exploration and the development of alternative raw materials. Intensifying research efforts to enhance pectin yield and quality, while identifying new high-quality sources is essential. By prioritizing green and efficient production practices, the pectin industry can develop sustainably, while meeting increasing market demands.

In conclusion, advancing the study and application of pectin through innovative extraction methods and alternative raw materials will not only strengthen the industry’s resilience but also positively contribute to environmental and economic sustainability.

## Figures and Tables

**Table 1:** Pectin contents varied from different plant materials.

Source	Pectin Content/%	Reference
Orange Peel	20–30	[3]
Banana Peel	1.0–1.5	[5]
Carrot	10–15	[8]
Apple Pomace	10–15	[9,10,11]
Beet Pulp	20–25	[12]
Potato Pomace	8–10	[13,14]
Pineapple Peel	2.4–4.6	[15,16]
Sunflower Disk	11.2	[17]
Pomelo Peel	15–30	[18]
Sweet Potato Peel	10–30	[19]
Dragon Fruit Peel	3.6–6.2	[20,21]
Hawthorn	17.7	[22]
Watermelon Peel	15–25	[23,24]
Tofu Tree Leaves	15–25	[25]
Grape Pomace	2.8–11.0	[26,27]
Jackfruit Peel	29	[28]

**Table 2:** Comparison of pectin extraction methods.

Category	Treatment	Source	Yield	Advantages	Disadvantages	Reference
Physical Methods	Microwave	Watermelon	16.2%	High efficiency, short time, energy saving, low solvent usage, simple operation	Not suitable for large materials, strict time control required	[24]
	Ultrasound	Orange Peel	30.59%	Reduces intermolecular forces, promotes pectin extraction, high-quality pectin with good color	Easy depolymerization and degradation, complex extraction process, higher cost	[31]
	Supercritical CO_2_	Pomelo Peel	22.64%	Strong continuous production capability, short extraction time, no solvent residue	Complex equipment, higher cost	[32]
	High Pressure Pulse	Okra	17.62%	Strong penetration, low energy consumption, continuous production possible	Complex equipment, limited application scenarios	[33]
	Steam Explosion	Passion Fruit Peel	10.7%	High processing efficiency, good results, significantly reduces energy and cost, reduces harmful chemical production	Difficult to control the uniformity of raw material treatment, pectin hydrolysis in high temperatures	[34]
	Subcritical Water	Persimmon Peel	7.62%	High extraction efficiency, low energy consumption, good selectivity, high product purity	Complex operation steps, high temperature and pressure can cause component denaturation or hydrolysis	[35]
Chemical Methods	Acid Extraction	Durian Peel	12.12%	Simple process, easy to operate, low cost	Destroy pectin structure, affecting pectin quality, unfriendly to the environment and equipment	[36]
	Ion Exchange	Apple Peel	26.26%	Reduces intermolecular forces, promotes pectin extraction, high-quality pectin with good color	Prone to depolymerization and degradation, complex extraction process, higher cost	[37]
Biological Methods	Enzymatic	Grape Peel	2.8%	Green and environmentally friendly, mild reaction conditions, strong enzyme specificity, high-quality pectin	Requires certain temperatures, high cost, low efficiency, not suitable for large-scale industrial production	[26]
	Microbial	Persimmon Peel	44.9%	Pollution-free, low consumption, relatively simple operation	Requires longer fermentation time	[38]

**Table 3:** Physicochemical properties of pectin.

Source	Extraction Method	Yield (%)	Esterification Degree (%)	Molecular Weight (kDa)	Pectin Type	References
Watermelon Rind	Citric Acid	24.30%	73.3	/	High Methoxyl	[23]
Grapefruit Peel	Acid Ethanol Precipitation	18.06%	68.3	746	High Methoxyl	[60]
Papaya Peel	Ion Exchange	22.00%	/	/	/	[52]
Durian Peel	Acid Ethanol Precipitation	2.59%	66.65		High Methoxyl	[60]
Pomegranate Peel	Acid Ethanol Precipitation	2.45%	67.75	/	High Methoxyl	[60]
Passion Fruit Peel	Acid Extraction	5.30%	74.50	/	/	[34]
Navel Orange Peel	Acid Extraction	9.16%	69.40	/	High Methoxyl	[60]
Sunflower Disk	Acid Extraction	6.06%	43.97	/	Low Methoxyl	[60]
	Ultrasonic Assisted	11.15%	34.06	175	Low Methoxyl	[17]
Persimmon Peel	Microbial Fermentation	0.449 g/g	62.51	/	High Methoxyl	[38]
	Subcritical Water Extraction	7.62%	40.61	21.79	High Methoxyl	[35]
Ramie Bast	Oxalic Ammonium Method	15.81%	53.11	/	/	[51]
Pumpkin	Oxalic Ammonium Method	6.09%	75.23	/	High Methoxyl	[50]
Dragon Fruit Peel	Dual-Phase Extraction	3.68%	/	/	/	[20]
Grape Pomace	Microwave Extraction	9.03%	82.29	/	/	[27]
Citrus Peel	Ultrasonic Assisted	17.00%	42.2	142	/	[4]
	Enzyme Method	11.93%	/	/	/	[56]
Banana Peel	Ultrasonic Method	3.20%	/	28.9	/	[2]
Apple Pomace	Acid Extraction	6.07%	38.26	/	/	[9]
	Ultrasonic Extraction	9.18%	83.2	/	High Methoxyl	[11]
